# Pathophysiological mechanisms of functional dyspepsia: a narrative review

**DOI:** 10.3389/fmed.2025.1624242

**Published:** 2025-11-10

**Authors:** Li Wang, YuPeng Shi, YaNi Li, XiaoJuan Wang, LiJuan Xu, Yue Zhang

**Affiliations:** Department of Gastroenterology, Honghui Hospital Affiliated to Medicine College of Xi'an Jiaotong University, Xi'an, Shaanxi, China

**Keywords:** functional dyspepsia, pathogenesis, gastrointestinal dynamics, immune response, psychosocial factors

## Abstract

**Background:**

Functional dyspepsia (FD) is a common gastrointestinal disorder with a global prevalence of about 40%. Its pathogenesis is multifactorial and not fully understood, which complicates diagnosis and treatment. Advances in research have improved understanding of the mechanisms underlying FD, offering opportunities to refine diagnosis and therapy.

**Summary:**

FD presents with upper abdominal discomfort, bloating, belching, and nausea, in the absence of structural disease. Its heterogeneous pathogenesis involves impaired gastric accommodation, delayed gastric emptying, mucosal immune activation, microbiota imbalance, altered neuroendocrine and sensory processing, and psychosocial influences. While treatment options have expanded, challenges remain due to overlapping symptoms and variability among patients.

**Key messages:**

FD is associated with abnormalities in gastrointestinal motility, immune responses, brain–gut interactions, and psychosocial factors. Recognizing its heterogeneity is crucial for developing individualized management strategies. Better characterization of patient subtypes may improve diagnostic accuracy, guide therapy, and ultimately enhance clinical outcomes.

## 1 Introduction

Functional dyspepsia (FD), as one of the common functional gastrointestinal disorders (FGID), is characterized by postprandial fullness and discomfort, early satiety discomfort, upper and middle abdominal pain, and burning discomfort in the upper and middle abdominal area (1). FD is classified into upper Epigastric Pain Syndrome (EPS), Postprandial Distress Syndrome (PDS), and PDS overlapping EPS subtypes (2). The global prevalence of FD is high, with an overall prevalence of 10–40% in Western countries and 5–30% in Asian countries (3). Data from a regional survey in China showed (4) that the number of FD patients accounted for 36.7% of FGID outpatient visits. FD is characterized by a wide range of patients, recurrent symptomatic episodes, and is often accompanied by anxiety and depression, with a markedly reduced quality of life, and serious economic and social burdens due to frequent visits to the doctor, examinations, and treatments (4–6). FD is often accompanied by gastrointestinal symptoms overlapping with those of other FGID, and this group of patients may even be more likely than the primary gastrointestinal symptoms to suffer from FD. These patients often experience a greater number and severity of physical symptoms, along with higher healthcare utilization and a significantly reduced quality of life compared to those with primary gastrointestinal diseases (7–9). The pathogenesis of FD has not been fully elucidated, and the diversity of clinical manifestations has led to poor treatment outcomes for FD patients. In this paper, we present a review of the pathogenesis of FD, hoping to provide a reference for the selection of clinical treatment programs for FD (Table 1).

**Table 1:** Reported clinical features and abnormalities that may be present in some patients with functional dyspepsia (FD).

Sub-mechanism	Key features	Involved factors	Clinical relevance
Central nervous system regulation	- Reduction in gray matter volume and brain network dysfunction- Activation of the hypothalamic–pituitary–adrenal (HPA) axis under stress- Abnormal metabolism of neurotransmitters	5-HT, BDNF, dopamine, cortisol	Alters gastrointestinal motility, visceral sensitivity, and emotional regulation
Enteric neuromodulation	- Dysregulation of myenteric and submucosal plexuses- Imbalance of local neurotransmitters- Neuro-immune-microbiota axis disruption	ENS, 5-HT, VIP, cytokines, mast cells, short-chain fatty acids	Contributes to visceral hypersensitivity, dysmotility, and abnormal secretory function
Endocrine dysregulation	- Abnormal secretion of gastrointestinal hormones- Impact on gastric acid secretion, motility, and digestion	Gastrin, motilin, CCK, glucagon, somatostatin	Leads to symptoms such as early satiety, bloating, delayed gastric emptying

## 2 Abnormal gastrointestinal dynamics

### 2.1 Delayed gastric emptying

Gastric emptying is the process by which stomach contents pass through the pylorus into the duodenum, regulated by a complex interplay of neural and hormonal factors (10, 11). In some patients with functional dyspepsia (FD), delayed gastric emptying has been observed, and this may contribute to symptoms such as postprandial fullness and early satiety. However, numerous studies indicate that the association between delayed gastric emptying and clinical symptoms is weak, and true gastroparesis is relatively uncommon (12–15). Delayed emptying may result from various factors, including impaired gastric motility, hormonal dysregulation, and vagal dysfunction. Importantly, impaired accommodation and visceral hypersensitivity often coexist and may play a more prominent role in symptom generation. Therefore, delayed gastric emptying should be regarded as one of several possible mechanisms in FD, rather than a uniform explanation of its clinical presentation. Gastric dyskinesia refers to the weakened peristaltic and contractile function of the stomach, which is unable to effectively propel gastric contents into the intestine. Gastrointestinal hormones such as motilin, gastrin, cholecystokinin, and glucagon-like peptide-1 play important roles in the process of gastric emptying, and once these hormones are abnormally secreted they may affect the rate of gastric emptying (16). The vagus nerve has a key role in regulating gastric motility (17), and its dysfunction may lead to weakened gastric peristalsis, which in turn affects gastric emptying. In addition, abnormalities in the mechanical and sensory functions of the stomach may also be an important cause of delayed gastric emptying (18). Poor compliance of the gastric wall primarily results in impaired adaptive relaxation, leading to symptoms such as early satiety and postprandial fullness. Peristaltic dysfunction, by contrast, is characterized by abnormal gastric contractions that impair mixing and emptying. Although both accommodation and peristalsis are influenced by vagal pathways, they represent distinct mechanisms and should be considered separately in the pathophysiology of FD (19). The diagnosis of delayed gastric emptying usually relies on gastric emptying assays, such as isotope-labeled meal test and carbon 13-labeled breath test, which quantitatively assess the rate of emptying of gastric contents and provide a basis for clinical diagnosis and treatment (20, 21). Pharmacologic and non-pharmacologic treatments are important means of treating delayed gastric emptying. For pharmacological treatment, gastric stimulating drugs such as domperidone and mosapride help to accelerate the gastric emptying rate and alleviate the symptoms of dyspepsia by enhancing the peristaltic and contractile functions of the stomach (22). For non-pharmacological treatment, dietary modification, behavioral therapy, and psychotherapy are considered supportive approaches. Dietary adjustments, such as eating small and frequent meals and avoiding high-fat or high-fiber diets, may help alleviate symptoms in some patients (23, 24). Behavioral therapy and psychotherapy may improve quality of life and reduce symptom burden by addressing lifestyle factors and psychological stressors, which could indirectly influence gastric function (25, 26). In addition, treatments targeting hormonal abnormalities in the gastrointestinal tract have gradually attracted attention (27), such as improving gastric emptying by adjusting hormone secretion or action.

### 2.2 Fundus diastolic dysfunction

The gastric fundus mainly acts as a reservoir that accommodates ingested food. Mixing of gastric contents is primarily mediated by peristaltic and antiperistaltic movements of the corpus and antrum, which ensure proper breakdown and homogenization before emptying into the duodenum. Under normal conditions, when food enters the stomach, the gastric fundus undergoes a relaxation response to adapt to the entry of food, known as the adaptive relaxation response of the gastric fundus (28). This process is mediated by the vagus nerve, which promotes relaxation of the gastric fundus by releasing neurotransmitters such as nitric oxide (NO) (29). However, in patients with FD, gastric fundus diastolic dysfunction results in the failure of the gastric fundus to relax properly, and as a result, the ability to hold gastric contents is reduced, which in turn causes symptoms such as early satiety, bloating, and epigastric discomfort (1). The pathophysiologic mechanism of gastric fundus diastolic dysfunction involves multiple neural and muscular factors. First, vagal dysfunction is considered to be one of the main causes (30). In patients with FD, vagal activity may be diminished, resulting in a diminished adaptive relaxation response of the gastric fundus to food (31). In addition, gastric smooth muscle responsiveness to neurotransmitters may be reduced, which further exacerbates fundal diastolic dysfunction. Balemans et al. (32) showed that the gastrointestinal tract of patients with FD has an increased sensitivity to mechanical and chemical stimuli, and that this visceral hypersensitivity may lead to hyperalgesia to fundal dilatation, which may trigger symptoms in patients. Visceral hypersensitivity may be associated with abnormal processing of gastrointestinal signals by the central nervous system, which causes physiological discomfort in patients due to normal gastric dilatation. Maev et al. (33) found that low-grade inflammation was present in the gastric mucosa of patients with FD, and that this chronic inflammation may affect gastric neurological and muscular function, leading to impaired fundic relaxation. Damage to the gastric mucosal barrier may increase permeability to luminal contents such as food antigens and bacteria, triggering localized inflammatory responses and alterations in nerve function, ultimately contributing to impaired fundic compliance (34). Psychological factors may also play a role in fundic relaxation impairment.

### 2.3 Disorders of small bowel motility

As an important digestive and absorptive organ in the human body, the normal peristaltic function of the small intestine is crucial for food digestion and nutrient absorption (35). Small bowel dyskinesia is mainly manifested as a disruption of the rhythmicity and coordination of small bowel peristalsis, which leads to an abnormal rate of food transportation in the small bowel (36). Song et al. (37) showed that patients with FD may present with either excessive contraction or weakened peristalsis of the small bowel. Excessive contraction can increase intraluminal pressure and mechanical stimulation, contributing to abdominal discomfort or pain, whereas weakened peristalsis may impair mixing and propulsion of intestinal contents, leading to postprandial fullness and bloating. Both abnormalities may contribute to dyspeptic symptoms through different pathophysiological pathways. Interstitial Cells of Cajal (ICCs) play an important role in small intestinal motility disorders, and ICCs are considered the pacemaker cells of the gastrointestinal tract, responsible for regulating electrical activity and generating peristaltic waves (38). Abnormalities in ICCs, such as reduced numbers or impaired function, have been reported in FD and may contribute to motility disturbances (39). Factors including genetic mutations and inflammatory responses may influence ICC function (40). Although autonomic regulation interacts with ICC activity, direct evidence of ICC dysfunction being causally related to autonomic dysfunction in FD is lacking, and this association should be interpreted with caution. Patients with FD are often associated with hypersensitivity of the sensory nerves of the small intestine, which is an important factor contributing to the disturbance of small bowel motility (41). The sensory nervous system of the small intestine is responsible for monitoring the chemical and mechanical status of food and digestive juices and transmitting the information to the central nervous system to regulate peristaltic and secretory functions of the small intestine (42). Increased hypersensitivity of sensory nerves can lead to an overreaction to normal digestive activity, resulting in abnormal peristalsis. This increased sensory nerve hypersensitivity may be associated with localized inflammation, infection, or stress response (43). The small intestine has a rich microbiota, which is a participant in the maintenance of normal small intestinal function. Brown et al. (44) found significant changes in the composition and function of the small intestinal microbiota in patients with FD, and this change may lead to impaired peristalsis in the small intestine. In addition, the reduction of microbial metabolites, such as short-chain fatty acids, may also affect small bowel motility. Psychological factors, such as chronic stress, anxiety, and depression, can influence small bowel motility by altering autonomic nervous system balance, increasing sympathetic activity, and reducing vagal tone. These changes may lead to either excessive or insufficient peristalsis, contributing to dysmotility symptoms observed in patients with functional dyspepsia (45, 46).

## 3 Gastrointestinal mucosa and immune response

### 3.1 Gastrointestinal inflammatory response

Recent studies have shown (47) that some degree of low-grade inflammation of the gastrointestinal mucosa exists in patients with FD, especially in the gastric and duodenal areas. Inflammatory responses have been suggested to contribute to the development of FD symptoms through mechanisms such as neuro-immune interactions, gastrointestinal dyskinesia, and visceral hypersensitivity (48). These responses may be triggered by factors including infection, food intolerance, or immune dysregulation, although the strength of these associations remains under investigation. *Helicobacter pylori* (*H. pylori*) infection has frequently been studied in this context, and it is detected in a substantial proportion of FD patients (49, 50). However, not all FD patients exhibit such changes, and the causal relationship remains debated. The infection of this bacterium can cause chronic inflammation of the gastric mucosa and release a series of cytokines and chemokines, such as interleukin-1β (IL-1β), interleukin-6 (IL-6), and tumor necrosis factor-*α* (TNF-α), etc., and these inflammatory mediators further activate the nerve endings in the gastrointestinal tract, leading to gastrointestinal dyskinesia and visceral hypersensitivity (51). In addition, *H. pylori* infection may also disrupt the gastric mucosal barrier function, making it easier for gastric acid and other harmful substances to enter the gastric wall, further aggravating the inflammatory response. In addition to *H. pylori*, food intolerance is also an important cause of gastrointestinal inflammatory response. Certain food components, such as lactose and gluten, may trigger an abnormal immune system response, leading to localized inflammation of the gastrointestinal mucosa. This localized inflammatory response can not only directly affect the function of the gastrointestinal tract, but also indirectly induce the development of FD symptoms by altering the structure of the intestinal flora and metabolites. For example, gluten intolerance can cause inflammation and villous atrophy in the small intestinal mucosa of patients with celiac disease, and although the majority of FD patients do not meet the diagnostic criteria for celiac disease, subclinical levels of gluten sensitivity are not uncommon in these patients (52). In addition, immune abnormalities in the gastrointestinal tract have been suggested to be an important part of the pathogenesis of FD. Biomed et al. (43, 53, 54) found that a large number of mast cells and T-lymphocytes were present in the gastrointestinal mucosa of patients with FD, and that aberrant activation and aggregation of these cells may be one of the causes of the inflammatory response in the gastrointestinal tract. By releasing histamine, prostaglandins, and other inflammatory mediators, mast cells not only directly stimulate the nerve endings of the gastrointestinal tract, but also lead to gastrointestinal dyskinesia by altering the contractile function of smooth muscle cells (55, 56). T lymphocytes, on the other hand, regulate the intensity and nature of local immune responses by secreting a variety of cytokines, thus exerting an influence on inflammatory responses in the gastrointestinal tract (57). During the pathophysiologic process of FD, the inflammatory response of the gastrointestinal tract not only directly affects the function of the gastrointestinal tract, but also alters the regulation of gastrointestinal tract function by the central nervous system through neuro-immune interactions (58, 59). Inflammatory responses in the gastrointestinal tract may activate local nerve endings and transmit inflammatory signals to the central nervous system, leading to an imbalance in the central nervous system’s regulation of the gastrointestinal tract. This imbalance may further exacerbate the symptoms of FD through abnormal activity of the hypothalamic–pituitary–adrenal axis (HPA axis) and the autonomic nervous system (46).

### 3.2 Role of *Helicobacter pylori* infection

*Helicobacter pylori* (*H. pylori*) is a Gram-negative, microaerobic, spiral-shaped bacterium that mainly parasitizes the submucosa of the stomach and can cause a variety of gastric diseases such as chronic gastritis, gastric ulcers, and gastric cancer (60). Rafi et al. (49) found that the prevalence of functional dyspepsia was significantly higher among individuals infected with *Helicobacter pylori* than those uninfected. However, as the study used symptom-based Rome criteria without endoscopic exclusion of organic disease, the overlap between FD and *H. pylori*-related dyspepsia could not be definitively excluded. Thus, this finding should be interpreted as an epidemiological correlation rather than causation. Prevalence was significantly higher than in uninfected individuals. The mechanisms by which *H. pylori* infection leads to FD include the following aspects. First, *H. pylori* infection causes chronic inflammatory response of gastric mucosa, which stimulates the body to release various inflammatory mediators and cytokines, destroys the barrier function of gastric mucosa, and leads to gastric dyskinesia and abnormal secretion of gastric acid, thus triggering FD symptoms (61). Secondly, *H. pylori* infection can affect the secretion of gastrointestinal hormones, such as gastrin and insulin, by altering the microecological environment of the gastrointestinal tract, thus affecting gastric emptying and peristalsis and leading to dyspepsia (62). In addition, *H. pylori* infection affects the sensory nerves of the gastric mucosa, increasing gastrointestinal sensitivity and causing patients to experience excessive pain and discomfort in response to normal gastrointestinal activities. The effect of *H. pylori* infection on gastric acid secretion is also one of the important mechanisms by which it leads to FD. *H. pylori* is capable of producing a variety of virulence factors, such as vacuolar toxin (VacA) and cytotoxin-associated gene A (CagA), which interfere with the normal secretion of gastric acid through multiple pathways (63). On the one hand, *H. pylori* infection can stimulate gastrin and gastric acid secretion; on the other hand, *H. pylori* infection directly reduces gastric acid secretion by destroying gastric mucosal cells. Abnormal secretion of gastric acid leads to retention of gastric contents and increase of pressure inside the stomach, thus triggering the symptoms of FD. H. pylori infection can also promote FD by affecting the immune response in the gastrointestinal tract. Suzuki et al. (64) showed that *H. pylori* infection can cause immune dysregulation of the gastrointestinal mucosa, leading to an overreaction to food and endogenous antigens in the gastrointestinal tract, which induces the symptoms of FD. *H. pylori* infection can also lead to motility disorders and sensory hypersensitivity in the gastrointestinal tract by inducing abnormal activity in the gastrointestinal nervous system, causing pain and discomfort to normal gastrointestinal activities. Although *H. pylori* infection plays an important role in the pathogenesis of FD, not all patients with FD are accompanied by *H. pylori* infection. Adadi et al. (65) showed that eradication of *H. pylori* significantly relieved the symptoms of some patients with FD, but the prevalence is still controversial. Therefore, in clinical practice, whether or not to perform *H. pylori* testing and treatment for FD patients should be considered comprehensively according to the specific conditions of patients. Although *H. pylori* eradication may improve symptoms in a subset of patients with functional dyspepsia, meta-analyses have shown that the clinical benefit is modest, with only about 1 in 4 patients reporting meaningful symptom relief following eradication therapy (66). In addition, further research on the specific mechanism of *H. pylori* infection in the pathogenesis of FD is needed to provide a more scientific basis for clinical treatment.

### 3.3 Intestinal microecological imbalance

The intestinal microcosm is a complex ecosystem composed of a large number of microorganisms, including bacteria, fungi, viruses, and protozoa. These microorganisms maintain the stability and balance of the intestinal environment through interactions and symbiotic relationships in the human gut. Under normal conditions, a dynamic equilibrium is formed between gut microbes and the host, which together promote digestion, absorption and immune function. However, when this balance is disrupted, it leads to an imbalance in intestinal microecology, which in turn triggers FD. Vitellio et al. (67) reported that some FD patient groups exhibit alterations in gut microbiota composition, including a relative reduction in beneficial bacteria (e.g., Lactobacillus and Bifidobacterium) and an increase in potentially harmful bacteria (e.g., *Escherichia coli* and anaerobes). These findings suggest possible links between dysbiosis and gastrointestinal dysfunction. However, such changes are not universal among all FD patients, and significant heterogeneity exists across studies. Therefore, further research is needed to identify which patients truly have abnormal microbiota and how these alterations relate to symptom generation. On the one hand, the reduction of beneficial bacteria in the intestinal tract will weaken its inhibitory effect on pathogenic bacteria, allowing pathogenic bacteria to proliferate and produce a variety of harmful metabolites, destroying the intestinal mucosal barrier and increasing intestinal permeability (68). On the other hand, an increase in harmful bacteria can lead to an increase in intestinal inflammatory response, triggering abnormal activation of the intestinal nervous system, which can lead to intestinal motor dysfunction and sensory hypersensitivity (69). Gut microecological imbalance also exacerbates the symptoms of FD by affecting the function of the gut-brain axis. The gut-brain axis is a complex network of interactions between the gut and the central nervous system through neural, endocrine, and immune pathways (70). Gut microbes can influence brain function and behavior through pathways such as metabolites, neurotransmitters, and immune factors. Similarly, the central nervous system can regulate the function of the gut through the autonomic and hypothalamic–pituitary–adrenal axes. In patients with FD, intestinal microecological imbalances can lead to bi-directional dysregulation of the gut-brain axis, which can exacerbate the symptoms of dyspepsia (71). This is manifested in a series of clinical symptoms such as abnormal intestinal motility, delayed gastric emptying, and hypersensitivity of gastrointestinal sensation. Gut microecological imbalance is also closely related to the psychological state of FD patients. Huang et al. (72) found that the incidence of anxiety and depression was significantly higher in FD patients than in the general population. Gut microbes influence the psychological state and behavioral responses of the host through the production of neurotransmitter precursors and the regulation of the intestinal nervous system and immune system. When the intestinal microecology is imbalanced, the metabolites of gut microbes, such as short-chain fatty acids and bile acids, can cross the blood–brain barrier and directly affect the function of the brain, leading to emotional and behavioral abnormalities (73). In addition, gut microbes can affect brain function by modulating the inflammatory response of the central nervous system, thus increasing the psychological burden of FD patients.

## 4 Neuroendocrine and sensory abnormalities

### 4.1 Central nervous system regulation

The central nervous system regulates gastrointestinal tract function through complex neural pathways and neurotransmitters. The brain-gut axis refers to the bidirectional communication between the central nervous system and the gastrointestinal tract, mediated by neural, endocrine, and immune pathways. Increasing experimental and clinical evidence supports its crucial role in functional gastrointestinal disorders, including FD, highlighting its relevance for both pathophysiology and potential therapeutic strategies (74). Neuroimaging studies have reported that some FD patients exhibit alterations in brain networks, which may be associated with abnormal gastrointestinal sensation and motility (75, 76). However, these findings are not consistent across all studies, and such changes are not present in all patients. Further research is needed to clarify which subgroups of FD patients demonstrate brain network alterations and how these relate to clinical symptoms. Wachowska-Kelly et al. (77) showed abnormalities in dopamine signaling pathways in FD patients, which may lead to gastrointestinal dysfunction. Brain-derived neurotrophic factor (BDNF) has also been suggested to play an important role in the pathogenesis of FD, and Ly et al. (78) found that the level of BDNF was significantly reduced in patients with FD, which may lead to abnormalities in the regulation of gastrointestinal tract function by the central nervous system. The regulation of the central nervous system involves not only the action of neurotransmitters, but also the influence of emotional and psychological factors. Mohajerani et al. (79) showed that FD patients have a higher prevalence of anxiety and depression, and that psychological stress affects gastrointestinal function through the central nervous system, which may exacerbate FD symptoms. Under stress, the body releases stress hormones such as cortisol, which affects the motor and secretory functions of the gastrointestinal tract through the hypothalamic–pituitary–adrenal axis (HPA axis). In addition, psychological stress can affect the sensory function of the gastrointestinal tract through the vagus nerve, leading to enhanced perception of normal physiological stimuli in the gastrointestinal tract, which in turn triggers or aggravates FD symptoms.

Recent studies (80, 81) indicate that psychotropic drugs may benefit FD patients by acting on the brain and modulating gastrointestinal function via the brain–gut axis. SSRIs increase synaptic 5-HT levels, thereby improving mood and anxiety while also influencing gastrointestinal motility and sensitivity (82). TCAs, such as amitriptyline, enhance the effects of neurotransmitters by inhibiting norepinephrine and 5-HT reuptake; they have been shown to reduce visceral hypersensitivity and ameliorate dysmotility (83). Benzodiazepines exert anxiolytic effects by enhancing GABA signaling in the CNS, which also contributes to the regulation of gastrointestinal motility and secretion (84). Collectively, these findings suggest that psychotropic drugs may alleviate FD symptoms not only through central effects on mood and anxiety, but also by modulating gastrointestinal function via the brain–gut axis (85, 86). Nevertheless, their precise mechanisms and clinical efficacy require further investigation.

### 4.2 Localized neuromodulation of the gastrointestinal tract

The nervous system in the gastrointestinal tract involves dual regulation by the Enteric Nervous System (ENS) and the Central Nervous System (CNS). The ENS is known as the “second brain” and has a complex neural network that regulates gastrointestinal functions independently, but is also influenced by the CNS (87). The enteric nervous system consists of two major plexuses: the Myenteric Plexus, which regulates the motor function of the gastrointestinal tract, and the Submucosal Plexus, which regulates secretion and blood flow (88). Delayed gastric emptying and abnormal small bowel peristalsis are common gastrointestinal motility disorders in patients with FD, both of which are closely related to the dysfunction of the intermuscular plexus. In addition, the submucosal plexus regulates the secretory activity of the gastrointestinal tract mucosa, and its abnormal function may lead to unbalanced gastric acid secretion, which in turn triggers dyspeptic symptoms (89). Local neuromodulation of the gastrointestinal tract also includes the regulation of visceral sensitivity. FD patients often show increased sensitivity to stimuli such as gastrointestinal dilatation and pressure, and this visceral hypersensitivity is closely related to abnormal discharge and neurotransmitter imbalance in the enteric nervous system (90). Liang et al. (91, 92) reported that altered levels of neurotransmitters such as 5-hydroxytryptamine (5-HT) and vasoactive intestinal peptide (VIP) have been observed in some FD patients. However, findings remain heterogeneous and inconsistent across studies, and these alterations are not universal. While 5-HT and VIP are known to play important roles in regulating gastrointestinal motility and sensory functions, further research is needed to clarify their specific involvement in FD pathophysiology. 5-HT is not only involved in the regulation of gastrointestinal motility, but also affects mood and pain perception through its interactions with the central nervous system, whereas VIP affects the digestive process mainly by regulating gastrointestinal blood flow and secretory functions. In addition, local neuromodulation of the gastrointestinal tract is influenced by the immune system. Immune cells in the gut interact with the enteric nervous system by releasing signaling molecules, such as cytokines, which influence gastrointestinal function (93). Patients with FD are often associated with a mild chronic inflammatory response, and this inflammatory state may lead to abnormal motor and sensory function in the gastrointestinal tract by altering the release of neurotransmitters and neuronal excitability. Vicentini et al. (94) found that the microbiota also plays an important role in the local neuromodulation of the gastrointestinal tract. The gut microbiota directly or indirectly influences the function of the enteric nervous system through the production of short-chain fatty acids, neurotransmitters, and other metabolites. Some studies have reported alterations in the gut microbiota of subsets of FD patients, suggesting a potential link between dysbiosis and disturbances in the neuro–immune–microbial axis that may influence gastrointestinal function (95). However, these findings are inconsistent, and current evidence is insufficient to conclude that such alterations are present in all patients.

### 4.3 Impact of endocrine factors

In recent years, the influence of endocrine factors in FD has gradually gained attention. Yakabi et al. (96) showed that gastrointestinal hormones play an important role in regulating the function of the digestive tract, and the abnormal secretion or dysfunction of these hormones may be one of the important causes of the pathogenesis of FD. A number of gastrointestinal hormones, including gastrin, glucagon, cholecystokinin, gastric motility, gastric depressin, and growth inhibitor, showed abnormal secretion to varying degrees in FD patients. First, gastrin plays a key role in gastric acid secretion and gastrointestinal motility, and abnormal gastrin levels in FD patients may lead to disorders in gastric acid secretion, which may cause symptoms such as epigastric discomfort and early satiety and bloating (97). Secondly, as an important gastrointestinal hormone, glucagon, by stimulating pancreatic secretion of pancreatic juice, regulating gastric acid neutralization and the digestive and absorptive processes in the small intestine, abnormal levels of glucagon may lead to pancreatic dysfunction, which further aggravates the symptoms of FD (98). In addition, cholecystokinin plays an important role in regulating bile secretion and fat digestion and absorption, and abnormal cholecystokinin secretion in FD patients may lead to poor fat digestion and absorption, triggering symptoms such as abdominal distension and abdominal pain (99). Abnormal secretion of gastrin, a key hormone in regulating gastrointestinal peristalsis, may lead to delayed gastric emptying and gastrointestinal motility dysfunction, which may cause aggravation of the symptoms of FD (100). Gastrin and growth inhibitor, on the other hand, play important roles in inhibiting gastric acid secretion and the regulation of gastrointestinal hormones, and their abnormal secretion may also be one of the potential mechanisms for the pathogenesis of FD.

## 5 Psychosocial factors

### 5.1 Effects of stress and emotions on digestive functioning

Stress and emotional changes significantly affect the functioning of the digestive tract through the central nervous system. In some patients, *H. pylori* infection or stress-related physiological changes may increase acid secretion, aggravating epigastric pain or burning symptoms consistent with the EPS (epigastric pain syndrome) subtype. In other patients, chronic mucosal injury or autonomic imbalance may reduce acid secretion, impair digestion, delay gastric emptying, and lead to symptoms resembling the PDS (postprandial distress syndrome) subtype. Moreover, activation of the sympathetic nervous system during stress may slow intestinal peristalsis and modulate acid secretion, contributing to dyspeptic symptoms in some, but not all, patients (101). At the same time, stress and negative emotions stimulate the activity of the hypothalamic–pituitary–adrenal (HPA) axis and secrete a large amount of stress hormones such as cortisol, which not only directly affect the motor function of the gastrointestinal tract, but also further aggravate the symptoms of FD by altering the distribution of blood flow in the gastrointestinal tract and the function of the mucosal barrier (102). Second, long-term psychological stress and mood swings can lead to dysregulation of the gastrointestinal microbiota. Karl et al. (103) showed that stress can alter the composition and metabolites of the intestinal flora, leading to an increase in intestinal permeability, which in turn induces intestinal inflammation and immune responses, and all of these changes negatively affect digestive function. In addition, psychological stress and negative emotions can affect the secretion of gastrointestinal hormones. Hormones such as gastrin, gastric actin, and cholecystokinin play an important role in peristalsis and digestion in the gastrointestinal tract, whereas stress and negative emotions inhibit the normal secretion of these hormones, leading to gastrointestinal dysfunction (104). In addition, emotional factors can exacerbate the subjective perception of symptoms by influencing the threshold of visceral susceptibility, which enhances the patient’s perception of normal physiologic activity in the gastrointestinal tract (105). This visceral hypersensitivity is prevalent in FD patients, suggesting that psychological factors play an important role in the development of the disease. Finally, stress and emotional problems are often accompanied by poor lifestyle and behavioral habits, such as irregular diet, smoking, and alcohol abuse, which likewise adversely affect gastrointestinal function and further aggravate the symptoms of FD (106, 107). Psychosocial factors, including anxiety, depression, somatization, and adverse life events, have been shown to exacerbate the onset and persistence of FD symptoms. In addition, lifestyle-related elements such as circadian rhythm dysregulation and sleep disturbance may indirectly influence FD pathophysiology by contributing to autonomic imbalance and neuroendocrine disruption. These associations underline the importance of a multidimensional approach that considers both biological and psychosocial domains in the management of FD.

### 5.2 The role of psychological factors in the pathogenesis of FD

Esterita et al. (5) showed that psychosomatic factors such as anxiety, depression, and stress are closely related to the development of FD. Psychology affects GI tract function through multiple pathways, which leads to the appearance of FD symptoms. On the one hand, psychological factors affect the motor function of the gastrointestinal tract through the brain-gut axis. When an individual faces stress or mood swings, sympathetic nervous system activity is increased and vagal function is inhibited, which in turn affects gastrointestinal motility and gastric emptying function (108). In addition, psychological stress can lead to activation of the hypothalamic–pituitary–adrenal axis (HPA axis), resulting in increased secretion of stress hormones such as cortisol, which can directly or indirectly affect gastrointestinal tract function and lead to exacerbation of FD symptoms (109). On the other hand, psychological factors can also lead to the development of visceral hypersensitivity by affecting the sensory function of the gastrointestinal tract. Visceral hypersensitivity is a common pathophysiological phenomenon in patients with FD, which refers to patients’ excessive pain or discomfort in response to normal physiological stimuli in the gastrointestinal tract (110). Oudenhove et al. (111) found that negative emotions, such as anxiety and depression, increase the central nervous system’s sensitivity to gastrointestinal sensory signals and lower the pain threshold, resulting in exaggerated responses to gastrointestinal pressure, distension, and other stimuli, manifesting as visceral hypersensitivity. In addition, psychological factors can affect the microecological balance of the gastrointestinal tract by altering the immune function and inflammatory response of the gastrointestinal mucosa. Long-term psychological stress and negative emotions can lead to impaired mucosal barrier function in the gastrointestinal tract, making it easier for harmful substances to pass through the mucosal barrier, triggering local inflammatory responses and possibly affecting the balance of gastrointestinal flora, thus exacerbating the symptoms of FD (112). Lee et al. (113) demonstrated that the role of psychological factors in the pathogenesis of FD does not exist in isolation, but rather, it interacts with other pathophysiological factors that together lead to the onset and development of FD.

### 5.3 Influence of lifestyle and dietary habits

Unhealthy eating habits are one of the important factors leading to FD. In modern society, many people often consume high-fat and high-sugar foods due to busy work and unhealthy lifestyle habits, which not only increase the gastrointestinal burden but may also trigger gastrointestinal inflammatory responses, thereby contributing to dyspeptic symptoms (114). In addition, irregular diet is also an important cause of FD. Xu et al. (115) showed that long-term irregular meals can lead to gastric acid secretion disorders and abnormal gastrointestinal dynamics, which in turn cause symptoms of dyspepsia such as epigastric discomfort and a feeling of fullness. Bad habits such as smoking and drinking alcohol can also negatively affect the digestive system (107). Smoking not only affects gastric acid secretion, but also impairs the gastric mucosal barrier function, increases gastrointestinal sensitivity, and induces dyspeptic symptoms. Alcohol, on the other hand, further aggravates dyspepsia by directly stimulating the gastrointestinal mucosa, leading to gastrointestinal inflammatory response and dysfunction. Lack of exercise is also one of the important factors leading to FD. Ismail et al. (116) showed that appropriate physical exercise can promote gastrointestinal peristalsis, increase gastrointestinal motility, and improve digestive function, whereas a sedentary lifestyle leads to a slowing down of gastrointestinal peristalsis and a delay in gastric emptying, which in turn leads to dyspepsia.

## 6 Other host-associated factors

### 6.1 Impact of sleep disorders

Both decreased sleep quality and decreased sleep duration are closely associated with the development of functional dyspepsia. Orjatsalo et al. (117) showed that chronic sleep deprivation leads to autonomic dysfunction and increased sympathetic excitability, which affects normal peristalsis of the gastrointestinal tract and gastric acid secretion. Specifically, sleep disorders can adversely affect gastrointestinal function through multiple pathways. First, sleep deprivation and poor sleep quality can lead to impaired gastrointestinal tract motility. Normally, peristalsis of the gastrointestinal tract is regulated by nerves and hormones, while sleep disorders can interfere with this process, leading to weakened peristalsis and delayed gastric emptying, which in turn can cause symptoms of indigestion such as bloating and a feeling of fullness. Secondly, sleep disorders also affect the secretion of gastric acid. Schey et al. (118) found that long-term sleep deprivation leads to increased secretion of gastric acid and excessive acidity in the stomach, which is easy to damage the gastric mucosa and cause gastrointestinal disorders such as gastritis, further aggravating the symptoms of dyspepsia. In addition, the relationship between sleep disorders and functional dyspepsia is also reflected in the regulatory mechanism of the brain-gut axis. The brain-gut axis refers to the bidirectional information transfer pathway between the brain and the gastrointestinal tract, and sleep disorders can increase gastrointestinal sensitivity by affecting the function of the brain-gut axis. Specifically, patients with sleep disorders are more likely to feel discomfort and pain in the gastrointestinal tract, which may be due to the enhanced response of the central nervous system to gastrointestinal stimuli (119). Psychological factors also play an important role in this, as long-term sleep disorders can lead to negative emotions such as anxiety and depression, which have an impact on gastrointestinal function through the brain-gut axis, creating a vicious cycle (120). Notably, the effects of sleep disorders on functional dyspepsia are not limited to physiological mechanisms, but also involve changes in behavioral habits. Keshteli et al. (121) showed that sleep deprivation is often accompanied by unhealthy lifestyles, such as irregular diets and lack of physical activity, which in themselves can be predisposing factors for functional dyspepsia. Therefore, improving sleep quality is important for the prevention and alleviation of functional dyspepsia. Treating sleep disorders also has a positive effect on the management of functional dyspepsia. Clinically, methods to improve sleep quality include sleep hygiene education, cognitive behavioral therapy, and medication. Sleep hygiene education aims to improve patients’ sleep quality through measures such as adjusting work and rest schedules and creating a good sleep environment; cognitive behavioral therapy reduces sleep-related anxiety by changing patients’ negative perceptions of sleep (122–124); and medication mainly includes the use of sleep aids and anti-anxiety medications to alleviate sleep disorders and related symptoms in patients.

### 6.2 Effects of circadian rhythms

Circadian rhythm, or biological clock, refers to the physiological and behavioral changes of an organism during a 24-h cycle, and this rhythm is regulated by the biological clock genes located in the suprachiasmatic nucleus (SCN) of the hypothalamus, which influences a variety of physiological functions including the gastrointestinal tract (125). Malloy et al. (126) demonstrated that disruption of circadian rhythms is one of the most important predisposing factors for functional dyspepsia. First, circadian rhythm is an influential factor in gastrointestinal dynamics. Under normal circumstances, peristalsis and emptying of the gastrointestinal tract show regular changes between day and night, and the gastrointestinal tract is more active during the day, which is favorable to the digestion and absorption of food, while the gastrointestinal tract activity is weakened at night, which is mainly for repair and maintenance. However, circadian rhythm disorders can lead to abnormalities in gastrointestinal dynamics, manifested as delayed or rapid gastric emptying, uncoordinated intestinal motility, etc., which can lead to the symptoms of functional dyspepsia (127). Kim et al. (128) found that the incidence of functional dyspepsia was significantly higher in the population of night shift workers and time travelers than that of people with regular work routines, which was closely related to the disorders of their circadian rhythms. Secondly, circadian rhythms also play an important role in regulating gastric acid secretion. Gastric acid secretion also has a circadian rhythm, usually less at night and more during the day, especially after meals, to help digest food. Disturbance of circadian rhythm will lead to irregularity of gastric acid secretion, which may result in increased secretion of gastric acid at night and insufficient secretion of gastric acid during the day, which will not only directly stimulate the gastric mucosa and cause gastric discomfort, but will also affect the normal digestion of food, resulting in bloating, nausea and other indigestion symptoms (129). In addition, the disturbance of gastric acid secretion may also lead to gastroesophageal reflux disease (GERD), further aggravating the symptoms of functional dyspepsia. In addition, circadian rhythms have an important impact on the repair and protective mechanisms of the gastrointestinal mucosa. Pagel et al. (130) showed that the repair of the gastrointestinal mucosa mainly takes place at night, and this process is regulated by circadian rhythms. Especially in patients with functional dyspepsia, the gastrointestinal mucosa itself may have some damage, which can be further aggravated by circadian rhythm disruption. In addition, circadian rhythms affect gastrointestinal function by regulating the neuroendocrine system. Gastrointestinal function is not only controlled by the autonomic nervous system, but also regulated by a variety of hormones, such as gastrin, insulin and cortisol. Circadian rhythm disruption will break the balance of hormone secretion, thus affecting the normal function of the gastrointestinal tract. For example, cortisol, as an important stress hormone, has a clear circadian rhythm in its secretion, which usually peaks in the morning and declines at night (131). If the circadian rhythm is disturbed, the rhythm of cortisol secretion is disrupted, which may lead to an over- or under-response to stress and affect the normal function of the gastrointestinal tract. Finally, circadian rhythm disruption may also indirectly contribute to the development of functional dyspepsia by affecting the balance of intestinal flora. Intestinal flora plays an important role in maintaining the health of the gastrointestinal tract, and its composition and function also have circadian rhythms. Zhao et al. (132) found that circadian rhythm disruption increases the proportion of harmful bacteria, which can disrupt the ecological balance of the intestine, and trigger or exacerbate the symptoms of functional dyspepsia.

To summarize, circadian rhythm affects gastrointestinal function in many ways, including gastrointestinal dynamics, gastric acid secretion, gastrointestinal mucosal repair, neuroendocrine regulation, and intestinal flora balance. Circadian rhythm disruption has been reported to be associated with functional dyspepsia. Improving sleep–wake habits and restoring a normal circadian rhythm may help prevent or alleviate dyspeptic symptoms, although further research is needed to confirm its causal role. However, due to the complexity of circadian rhythms on gastrointestinal function, further in-depth studies are needed to fully reveal the mechanism of circadian rhythms and provide a more scientific basis for the prevention and treatment of functional dyspepsia.

## 7 Clinical implications and practical guidance

FD is characterized by substantial pathophysiological heterogeneity, and current evidence indicates that no single abnormality can fully account for the clinical phenotype. Nevertheless, several mechanistic domains have demonstrated relatively higher diagnostic and therapeutic relevance, including impaired gastric accommodation, delayed gastric emptying, visceral hypersensitivity, and altered brain–gut axis signaling. From a clinical perspective, a rational approach should be structured around a tiered evaluation model. The initial step involves systematic exclusion of organic disease through endoscopic and histopathological assessment when indicated, in order to avoid diagnostic confounding. Subsequently, targeted functional investigations such as scintigraphic or breath-test assessment of gastric emptying, nutrient drink testing for gastric accommodation, and validated questionnaires for visceral hypersensitivity can delineate dominant pathophysiological processes. In parallel, psychometric evaluation of anxiety, depression, and sleep disorders is essential, as these factors are established modulators of central processing of visceral signals and significantly alter symptom severity.

Therapeutically, clinical management should prioritize individualized matching between dominant pathophysiological abnormalities and corresponding interventions. Prokinetic agents may be considered in patients with documented delayed gastric emptying, fundus-relaxing drugs in those with impaired accommodation, and centrally acting neuromodulators such as tricyclic antidepressants or SSRIs in patients with prominent visceral hypersensitivity and comorbid affective disturbance. Circadian rhythm dysregulation should be regarded as an associated pathophysiological factor rather than a primary etiology; mechanistic evidence supports its role in altering autonomic balance and neuroendocrine output, but its clinical weighting remains to be clarified. From a translational research perspective, priority should be assigned to elucidating mechanistic links between impaired gastric accommodation, sensory hypersensitivity, and aberrant central processing, particularly using multimodal approaches integrating high-resolution manometry, brain imaging, and microbiota–immune–neural interaction profiling.

## 8 Summary

Notably, among the various pathophysiological mechanisms discussed, the most strongly supported by clinical and experimental evidence are impaired gastric motility (e.g., delayed gastric emptying), *Helicobacter pylori* infection, visceral hypersensitivity, and neuroimmune alterations. Circadian rhythm disturbances and sleep disorders remain under investigation and are currently considered associative or modulatory rather than primary drivers; further studies are needed to clarify causal roles.

## Data Availability

The original contributions presented in the study are included in the article/supplementary material, further inquiries can be directed to the corresponding author.
